# A Sub-1 ppm/°C Reference Voltage Source with a Wide Input Range

**DOI:** 10.3390/mi15101273

**Published:** 2024-10-21

**Authors:** Yuchi Xiao, Chunlai Wang, Hongyang Hou, Weihua Han

**Affiliations:** 1 School of Physical Science and Technology, Lanzhou University, Lanzhou 730000, China; 220220939211@lzu.edu.cn (Y.X.); allan.w@bstsemi.com (C.W.); 220220938921@lzu.edu.cn (H.H.); 2 BASALT Semiconductor Co., Ltd., Wuhan 430000, China; 3 Guangzhou Institute of Blue Energy, Guangzhou 510555, China

**Keywords:** bandgap reference, wide input range, high-order curvature compensation, temperature coefficient

## Abstract

With the continuous advancement of electronic technology, the application of high-voltage integrated circuits is becoming increasingly prevalent in fields such as power systems, medical devices, and industrial automation. The reference circuit within high-voltage integrated circuits must not only exhibit insensitivity to temperature variations but also maintain stability across a broad voltage supply. This paper presents a bandgap reference (BGR) source capable of operating over a wide input range. This BGR employs a high-order curvature compensation method to eliminate nonlinear voltage terms, resulting in minimal temperature drift. The circuit achieves an impressive temperature coefficient (TC) of 0.88 ppm/°C over a temperature range from −40 °C to 130 °C. To ensure stable operation within a 4–40 V range, the design incorporates a pre-regulation circuit that stabilizes the supply voltage of the BGR core at a fixed value, thereby enhancing the ability to withstand variations in power supply voltage.

## 1. Introduction

Due to its low TC and high power supply rejection ratio (PSRR), the BGR voltage source, serving as a fundamental component in integrated circuits, has been extensively utilized in DC–DC converters, analog-to-digital converters, low-dropout regulators, and other mixed-signal applications [1,2,3]. However, the large variation in the supply voltage range can lead to fluctuations in the reference voltage provided by the BGR, consequently impacting circuit operation. High performance BGRs with a wide input range (e.g., 4–40 V) are extensively applied in high-voltage integrated circuits, including smart grids, energy storage systems, and electric vehicles [4].

In addition to supply independence, an ideal BGR should exhibit minimal sensitivity to temperature variations. A conventional BGR achieves a temperature-independent output by combining two voltages with opposite TCs [5]. Specifically, a complementary-to-absolute-temperature (CTAT) voltage comes from the base-emitter voltage $V_{\mathrm{BE}}$ of bipolar transistors, while the difference between the base-emitter voltages of two bipolar transistors operating at unequal current densities yields a directly proportional-to-absolute-temperature (PTAT) voltage. However, the presence of higher-order temperature term TlnT in the base-emitter voltage causes the output reference voltage to still fluctuate with temperature. As a result, the linear combination of positive and negative TC voltages often leads to a TC exceeding 10 ppm/°C. Previous studies have proposed various methods to mitigate the effects of the higher-order term TlnT. Lavrentiadis et al. employed MOSFET transistors operating in the sub-threshold region instead of bipolar transistors to generate reference voltage [6], achieving a TC of 24 ppm/°C. Du et al. [7] referenced the curvature compensation method from [8] to compensate for the TlnT nonlinearity; the author reported a typical TC of 1.5 ppm/°C but reached a current consumption of 267 μA. Various methodologies have been proposed by designers to generate a compensatory current that resembles the shape of the TlnT curve [9,10,11]. For example, Malcovati et al. utilized a $T^{2}$ current to compensate for nonlinear terms [9], while Cao and Shen et al. employed the emitter current and the base current of the transistor for compensation [10,11]. However, an analysis of the provided expressions for compensation current reveals that the compensation term does not adequately fit the nonlinear term TlnT, resulting in a temperature drift exceeding 1 ppm/°C.

**Figure 1:**
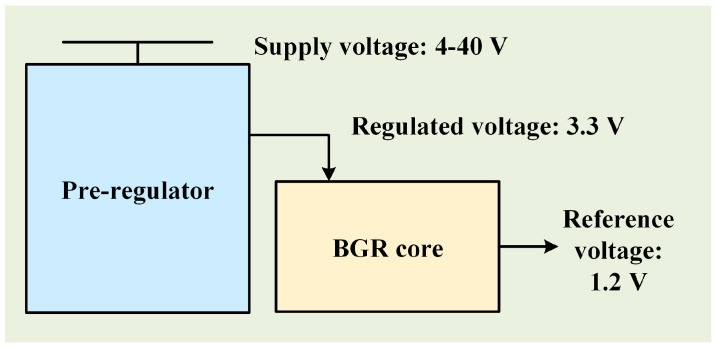
Block diagram of designed BGR.

This paper presents a BGR circuit designed to operate effectively over a broad voltage range. The proposed BGR incorporates advanced compensation techniques aimed at minimizing temperature drift. Experimental findings demonstrate that utilizing a 0.18 μm Bipolar-CMOS-DMOS (BCD) process, the reference voltage achieves a TC of less than 1 ppm/°C across a power supply range from 4 V to 40 V. To accommodate the wide-input voltage range required for the power supply and to enhance the precision of the BGR circuit, a customary approach involves first generating a low-voltage power supply using a pre-regulator circuit. This pre-regulated supply stabilizes voltage around 3.3 V, which subsequently powers the core BGR circuit. Within this wide-input range, the BGR core circuit produces a stable 1.2 V reference voltage with minimal TC. Figure 1 illustrates the block diagram of designed BGR.

## 2. Materials and Methods

### 2.1. Pre-Regulator Circuit

**Figure 2:**
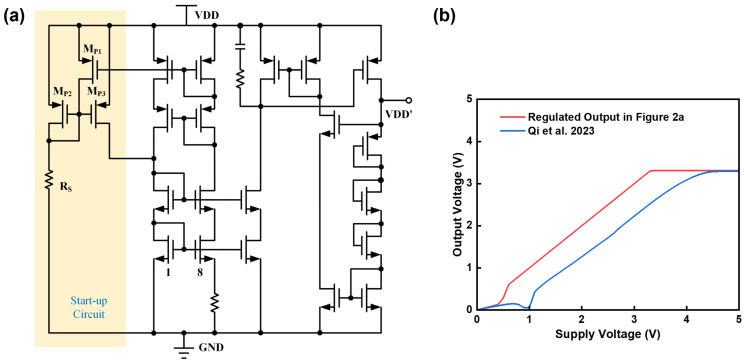
(**a**) Schematic of proposed pre-regulator circuit. (**b**) Regulated output voltages of two structures [11].

The pre-regulator circuit discussed in this article is illustrated in Figure 2a. Transistors $M_{P1}$, $M_{P2}$, $M_{P3}$ along with the resistor $R_{S}$ collectively form the start-up circuit, ensuring stable and reliable operation of the pre-regulator module. A self-biased current source is implemented using a cascode current mirror, providing a current that remains independent of the supply voltage. This current flows through the diode-connected MOS transistors to generate the regulated output voltage. High-voltage devices are utilized in the pre-regulator circuit to efficiently convert a wide range of input voltages into a lower, more stable output voltage. Incorporating a feedback loop minimizes the impact of power supply fluctuations on the output voltage stability. In contrast to the pre-regulator circuit described in [11], this design utilizes a PMOS transistor for driving rather than an NMOS transistor. This choice allows for a reduction in the minimum operating voltage of the pre-regulator circuit. As illustrated in Figure 2b, the PMOS-driven circuit achieves a lower minimum operating voltage by one threshold voltage compared to its NMOS-driven counterpart. During operation, the regulator maintains a tiny drop-out voltage relative to the supply, ensuring normal operation even under low-power conditions and reducing energy loss from the power source.

### 2.2. Nonlinearity in BGR

**Figure 3:**
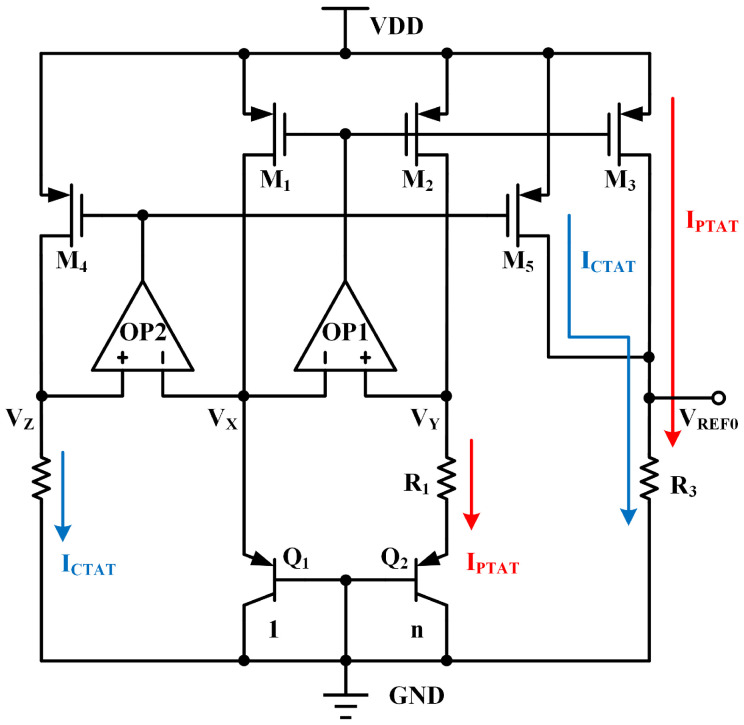
Schematic of a traditional current mode BGR.

Figure 3 presents a current-mode BGR circuit [12], where the operational amplifiers OP1 and OP2 adjust the gate voltage of the PMOS devices to equalize $V_{X}$, $V_{Y}$, and $V_{Z}$. Transistor $Q_{2}$ consists of n unit transistors connected in parallel, and $Q_{1}$ is a single unit transistor. This configuration generates PTAT and CTAT currents, with their expressions given as follows.

(1) $$I_{\mathrm{PTAT}}=\frac{V_{EB1}-V_{EB2}}{R_{1}}=\frac{V_{T}\ln n}{R_{1}}$$

(2) $$I_{\mathrm{CTAT}}=\frac{V_{EB1}}{R_{2}}$$

Here, $V_{EB1}$ and $V_{EB2}$ are emitter–base voltages of the bipolar junction transistor $Q_{1}$ and $Q_{2}$. $V_{T}=\mathrm{kT}/q$ is the thermal voltage, where k is the Boltzmann constant, T is the absolute temperature, q is the electron charge. The PTAT and CTAT currents are copied and passed through a resistor $R_{3}$ to generate a zero-TC voltage.

(3) $$\begin{array}{cc} V_{REF0} & =\left(I_{\mathrm{PTAT}}+I_{\mathrm{CTAT}}\right)\cdot R_{3}\\ & =\left(\frac{V_{T}\ln n}{R_{1}}+\frac{V_{EB1}}{R_{2}}\right)\cdot R_{3} \end{array}$$

However, the base–emitter voltage of a BJT is not a purely linear function of temperature; it can be expressed as [13]

(4) $$\begin{array}{cc} V_{BE}\left(T\right)= & V_{G}\left(T\right)-\left(\frac{T}{T_{r}}\right)V_{G}\left(T_{r}\right)+\left(\frac{T}{T_{r}}\right)V_{BE}\left(T_{r}\right)\\ \; & -\left(\eta -\delta \right)\left(\frac{kT}{q}\right)\ln\left(\frac{T}{T_{r}}\right) \end{array}$$

where $V_{G}\left(T\right)$ is the bandgap voltage at temperature *T*, η=4−n, *n* represents the order of temperature dependence of carrier mobility, δ is the order of temperature dependence of collector current. $T_{r}$ refers to reference temperature.

Equation (4) shows that besides a CTAT term, $V_{BE}\left(T\right)$ includes a $T\ln(T/T_{r})$ term, which introduces nonlinearity. The presence of this higher-order term causes the reference voltage to exhibit curvature as temperature varies. To achieve temperature independence of the reference voltage, it is necessary to eliminate this nonlinear term.

### 2.3. Implementation of High-Order Compensation

When high-precision reference voltages are required, the nonlinear terms in $V_{\mathrm{BE}}$ cannot be neglected. Exponential current generators have been employed in curvature compensation [14]. The compensation current is generated by sub-threshold MOS transistors, which exhibit significant deviations across different fabrication processes. The efficacy of this approach using a single compensation current is suboptimal, often necessitating multi-segment compensation for the circuit. Therefore, it is essential to generate a term that more accurately matches the nonlinear component.

**Figure 4:**
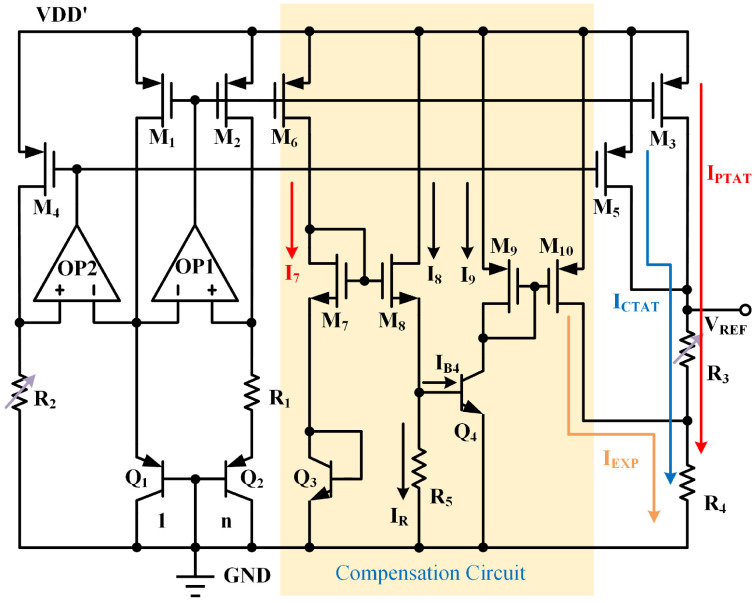
Proposed BGR core circuit with compensation.

Figure 4 shows the complete circuit of the proposed BGR core, based on the current-mode BGR circuit discussed in Section 2.2. In the circuit, transistors $M_{7}$ and $M_{8}$ have the same aspect ratio $W_{n}/L_{n}$. $M_{1}$, $M_{2}$, and $M_{6}$ have identical device dimensions. Bipolar junction transistors $Q_{3}$ and $Q_{4}$ consist of the same units in parallel. Currents for both PTAT and CTAT have been designed to be 100 nA. Since $V_{GS7}+V_{BE3}=V_{GS8}+V_{BE4}$, we have

(5) $$\begin{array}{cc} V_{GS7}-V_{GS8} & =V_{BE4}-V_{BE3}=V_{T}\ln\frac{I_{C4}}{I_{C3}}=V_{T}\ln\frac{I_{9}}{I_{7}}\\ \; & =\sqrt{\frac{2}{\mu_{n}C_{ox}\frac{W_{n}}{L_{n}}}}\left(\sqrt{I_{7}}-\sqrt{I_{8}}\right) \end{array}$$

where $V_{GS7}$ and $V_{GS8}$ are gate-source voltages of MOS transistors $M_{7}$ and $M_{8}$, $\mu_{n}$ represents the mobility of electrons, and $C_{ox}$ is the gate-oxide capacitance per unit area. The current flowing through $M_{8}$ can be expressed as

(6) $$\begin{array}{cc} I_{8} & =I_{R}+I_{B4}\\ \; & =\frac{V_{BE4}}{R_{5}}+\frac{1}{\beta }I_{9} \end{array}$$

where $V_{BE4}$ is the base–emitter voltage of bipolar junction transistor $Q_{4}$, β represents the common-emitter current gain of $Q_{4}$. Thus, we obtain the output current as follows:

(7) $$\begin{array}{cc} I_{9} & =I_{7}\cdot \exp\left(\frac{\sqrt{\frac{2}{\mu_{n}C_{ox}\frac{W_{n}}{L_{n}}}}\left(\sqrt{I_{7}}-\sqrt{I_{8}}\right)}{V_{T}}\right)\\ \; & =\frac{V_{T}\ln n}{R_{1}}\cdot \exp\left(\frac{\sqrt{\frac{2}{\mu_{n}C_{ox}\frac{W_{n}}{L_{n}}}}}{V_{T}}\left(\sqrt{\frac{V_{T}\ln n}{R_{1}}}-\sqrt{\frac{V_{BE4}}{R_{5}}+\frac{1}{\beta }I_{9}}\right)\right) \end{array}$$

**Figure 5:**
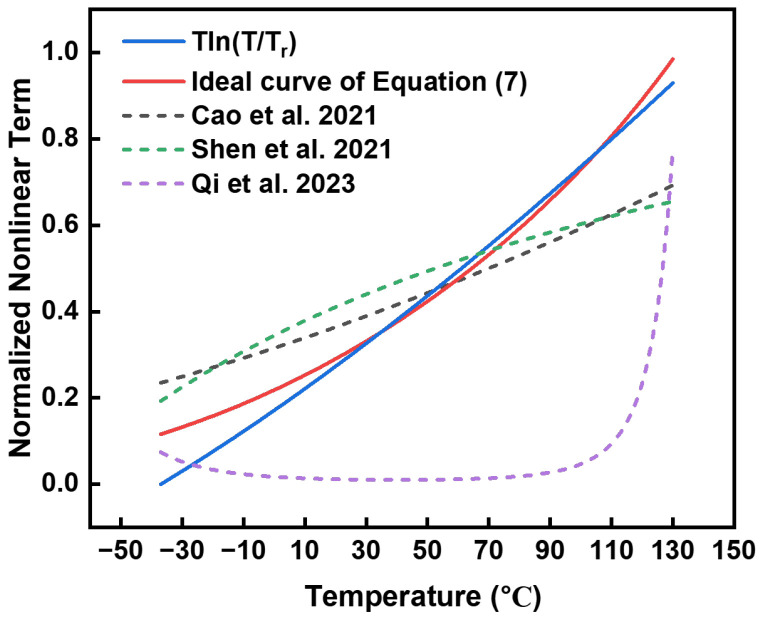
Normalized curve of $T\ln(T/T_{r})$, $I_{9}$, and compensation terms used in [9,10,11].

The resistor values of $R_{1}$ and $R_{5}$ can be determined through numerical simulations using specialized software (such as MATLAB R2023b). Nonlinear terms can be eliminated by applying compensating current. Figure 5 illustrates that compared with former research, the generated current effectively matches the $T\ln(T/T_{r})$ term across a wide temperature range. The error between these curves remains below 10% across temperatures ranging from 10 °C to 130 °C.

Transistor $M_{10}$ copies the current of $M_{9}$ to obtain the compensating current $I_{\mathrm{EXP}}$ and injects it into resistor $R_{4}$, thereby eliminating the nonlinear term and generating reference voltage.

(8) $$V_{REF}=\left(I_{PTAT}+I_{CTAT}\right)\cdot \left(R_{3}+R_{4}\right)+I_{EXP}\cdot R_{4}$$

Due to the errors and mismatches inherent in chip manufacturing, it is essential to adjust the resistance values in the circuit to minimize temperature drift caused by process variations. Notably, $R_{2}$ governs the linear trend of the reference voltage, while $R_{3}$ controls the magnitude of the output voltage. Resistor trimming networks are employed at $R_{2}$ and $R_{3}$ to modify these resistances.

Fuse trimming is a relatively conventional method for adjusting resistance values, where each resistor in a series resistor network is connected in parallel with a fuse. Each fuse is associated with two trimming pads, and prior to trimming, the fuse short-circuits the resistor. During the trimming process, a voltage pulse applied across the terminals of the fuse burns it out, gradually increasing the resistance value of the resistor network.

**Figure 6:**
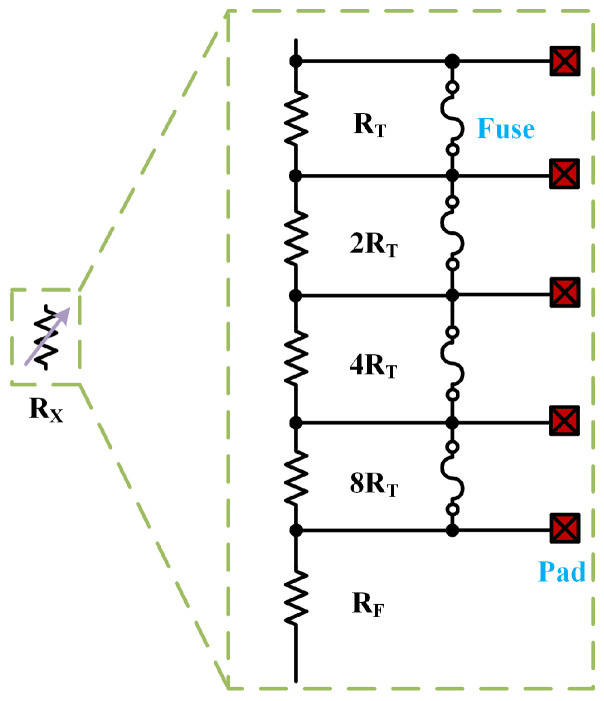
Resistance trimming network.

Figure 6 illustrates the resistance trimming network utilizing the fuse trimming technique employed in this work. The fixed resistance $R_{F}$ of $R_{2}$ and $R_{3}$ are 6.66 MΩ and 6.44 MΩ, respectively, and the unit trimming resistance $R_{T}$ connected in parallel with the fuse are 40 kΩ and 60 kΩ.

## 3. Results

**Figure 7:**
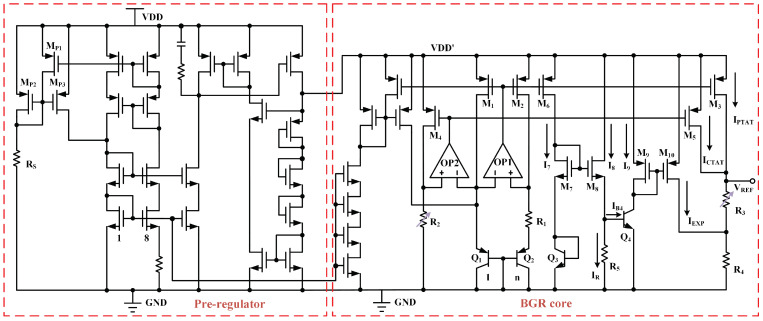
Proposed BGR structure.

**Figure 8:**
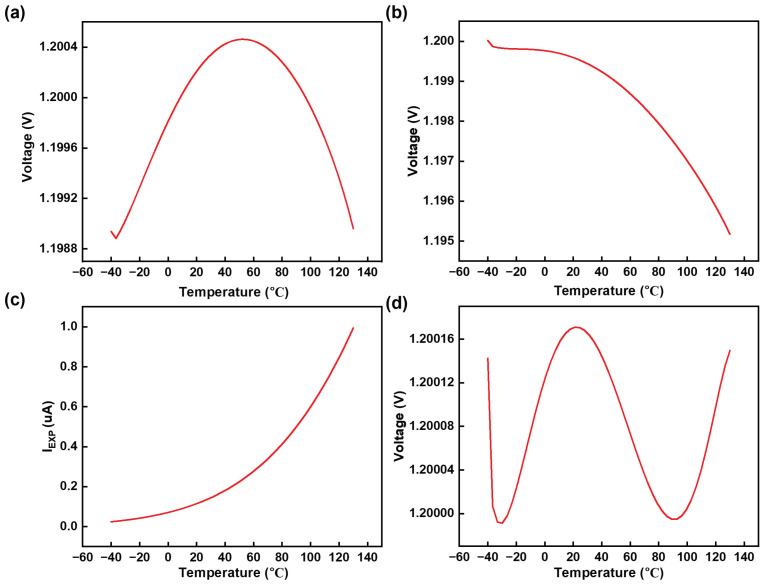
BGR output under temperature variation. (**a**) BGR output using structure in Figure 3. (**b**) BGR output without $I_{EXP}$. (**c**) Compensation current $I_{EXP}$. (**d**) BGR output after compensation.

The BGR is designed using a 0.18 μm BCD process. The schematic depiction of the overall circuit is illustrated in Figure 7. In the BGR core, we use the same start-up circuit as in the pre-regulator circuit. The mirrored current from the current source is injected into the BJT branch to help the circuit generate the reference voltage more quickly. The output of the BGR circuit is shown in Figure 8 across a temperature range of −40 to 130 °C. The output voltage from the structure depicted in Figure 3 exhibits a TC of 7.74 ppm/°C, as illustrated in Figure 8a. After implementing the advanced compensation methods proposed in this paper, the TC of the reference voltage was improved to 0.88 ppm/°C.

**Figure 9:**
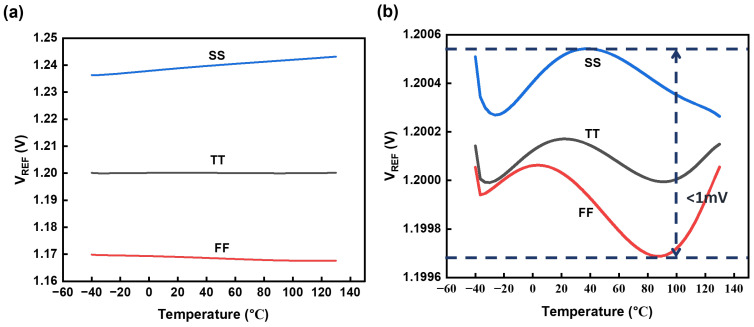
Output voltage with temperature across corners. (**a**) Reference without trimming. (**b**) Reference after trimming.

The untrimmed output of the BGR under different process corners is illustrated in Figure 9a (T, S, F are abbreviations for typical, slow, and fast, representing carrier mobilities. The first letter refers to NFETs, while the last letter refers to PFETs.). The variation between corners and temperatures reaches 6.5%. Through fuse trimming, the vertical discrepancy in output voltage across various corners is maintained within a margin of 1 mV. Table 1 presents the TCs of the proposed BGR before and after trimming. The results indicate that the circuit is capable of delivering a stable reference voltage despite significant temperature variations.

**Figure 10:**
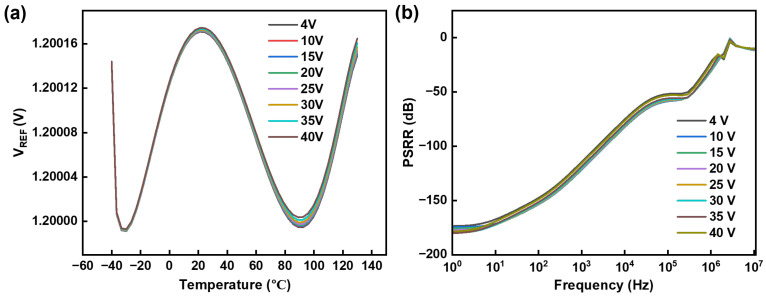
(**a**) Output voltage with temperature across various supply voltages. (**b**) PSRR across various supply voltages.

Figure 10a shows the output curves of the circuit at different supply voltages across varying temperatures. It can be observed that the changes in supply voltage result in an impact of less than 0.001% on the circuit output, which can be neglected. This observation is also supported by the PSRR curve depicted in Figure 10b. At a supply voltage of 4 V, the circuit achieves a PSRR of −167.38 dB at 10 Hz, −114.62 dB at 1 kHz, and −51.8 dB at 100 kHz. The results depicted in Figure 10 demonstrate that the circuit exhibits a robust resilience to power supply fluctuations.

Table 2 presents the performance of the BGR and compares it with other published results. Compared with other works, our design achieves the lowest TC. In addition to excellent temperature independence, our circuit also exhibits remarkable rejection of voltage variations. In [6], the use of MOSFETs operating in the sub-threshold region can reduce the power consumption of the circuit to some extent; however, the TC of the reference circuit will greatly increase.

## 4. Discussion

The proposed circuit not only achieves superior performance metrics but also demonstrates feasibility for practical applications requiring high precision and stability, such as power management systems and precision analog circuits. The advanced compensation techniques employed effectively address common challenges associated with temperature-induced variations, making this design a robust and reliable solution for modern integrated circuits.

However, compared to existing achievements, the minimum start-up voltage of our design remains relatively high, primarily constrained by the operating voltage of the BGR core. To further reduce the minimum operating voltage of the circuit, we could explore redesigning the BGR core module using processes characterized by lower threshold voltages and reduced static currents. This approach would also contribute to a decrease in the circuit’s power consumption. Future work could investigate further optimization and integration strategies to enhance performance and adaptability across different semiconductor processes.

## 5. Conclusions

This article presents a low-TC BGR circuit that employs an advanced high-order temperature compensation technique and can operate over a wide range of temperatures and supply voltages through the implementation of a pre-regulation module. With a minimum TC of 0.88 ppm/°C and a PSRR of −167.38 dB at 10 Hz, this circuit demonstrates good resilience against temperature and voltage variations.

## Figures and Tables

**Table 1:** Simulation TC under different corners.

Simulation Corner	TC Without Trimming	TC With Trimming
TT	0.88 ppm/°C	0.88 ppm/°C
FF	11.57 ppm/°C	1.84 ppm/°C
SS	32.42 ppm/°C	1.37 ppm/°C

**Table 2:** Performance summary and comparison.

	Design	[6]	[7]	[9]	[10]	[11]	[This Work]
Parameter							
Process (μm)		0.18	0.18 BCD	0.18 BCD	0.18 BCD	0.18 BCD	0.18 BCD
Supply voltage (V)		9–15	4.5–35	2.47–10	3–18	4.4–35	4–40
Reference (V)		2.507	2.5	1.218	2.048	1.205	1.2
Temperature (°C)		−20–80	−40–125	−50–125	−40–125	−40–125	−40–130
TC (ppm/°C)		24	1.5	9	1.2	3.83	0.88
Current consumption (μA)		8.47	267	-	279.6	23	9
PSRR (dB)		−60@10 Hz	−102.82@10 Hz	−86@120 Hz	-	−96@120 Hz	−167.38@10 Hz
		−53@1 kHz					−114.62@1 kHz
		−81@1 MHz					−51.8@100 kHz

## Data Availability

The original contributions presented in the study are included in the article. Further inquiries can be directed to the corresponding author.
